# Does ventilator-associated event surveillance detect ventilator-associated pneumonia in intensive care units? A systematic review and meta-analysis

**DOI:** 10.1186/s13054-016-1506-z

**Published:** 2016-10-24

**Authors:** Yunzhou Fan, Fang Gao, Yanyan Wu, Jie Zhang, Ming Zhu, Lijuan Xiong

**Affiliations:** Department of Nosocomial Infection Management, Union Hospital, Tongji Medical College, Huazhong University of Science and Technology, 1277 JieFang Avenue, Wuhan, 430022 China

**Keywords:** Ventilator-associated events VAE, Ventilator-associated pneumonia VAP, Surveillance, Meta-analysis

## Abstract

**Background:**

Ventilator-associated event (VAE) is a new surveillance paradigm for monitoring complications in mechanically ventilated patients in intensive care units (ICUs). The National Healthcare Safety Network replaced traditional ventilator-associated pneumonia (VAP) surveillance with VAE surveillance in 2013. The objective of this study was to assess the consistency between VAE surveillance and traditional VAP surveillance.

**Methods:**

We systematically searched electronic reference databases for articles describing VAE and VAP in ICUs. Pooled VAE prevalence, pooled estimates (sensitivity, specificity, positive predictive value (PPV), and negative predictive value (NPV)) of VAE for the detection of VAP, and pooled estimates (weighted mean difference (WMD) and odds ratio ([OR)) of risk factors for VAE compared to VAP were calculated.

**Results:**

From 2191 screened titles, 18 articles met our inclusion criteria, representing 61,489 patients receiving mechanical ventilation at ICUs in eight countries. The pooled prevalence rates of ventilator-associated conditions (VAC), infection-related VAC (IVAC), possible VAP, probable VAP, and traditional VAP were 13.8 %, 6.4 %, 1.1 %, 0.9 %, and 11.9 %, respectively. Pooled sensitivity and PPV of each VAE type for VAP detection did not exceed 50 %, while pooled specificity and NPV exceeded 80 %. Compared with VAP, pooled ORs of in-hospital death were 1.49 for VAC and 1.76 for IVAC; pooled WMDs of hospital length of stay were −4.27 days for VAC and −5.86 days for IVAC; and pooled WMDs of ventilation duration were −2.79 days for VAC and −2.89 days for IVAC.

**Conclusions:**

VAE surveillance missed many cases of VAP, and the population characteristics identified by the two surveillance paradigms differed. VAE surveillance does not accurately detect cases of traditional VAP in ICUs.

**Electronic supplementary material:**

The online version of this article (doi:10.1186/s13054-016-1506-z) contains supplementary material, which is available to authorized users.

## Background

Mechanical ventilation (MV) is a widely used intervention for critically ill patients in intensive care units (ICUs). Ventilator-associated pneumonia (VAP) is a clinically important, potentially preventable complication of mechanical ventilation [1–3].

Prior to 2013, the National Healthcare Safety Network (NHSN) monitored MV complications by VAP surveillance. The clinical diagnosis of VAP is based on clinical signs, chest radiography, and microbiological data. Clinical signs include: changes in sputum or tracheal secretions in terms of purulence, color, and/or increasing production; cough; temperature >38 or <36 °C; rales or bronchial breath sounds on examination, and worsening oxygenation. Laboratory findings include non-specific indicators of infection including leukocytosis (>12 × 10^9^ white blood cells (WBC)/L) or leukopenia (<4.0 × 10^9^ WBC/L). Signs on chest radiography include the development of new infiltrates or the presence of persistent and/or worsening infiltrates [4].

However, VAP surveillance relying on clinical criteria has proven highly problematic in practice, because most of these diagnostic criteria are not objective or specific [5–7], leaving a wide margin in the surveillance of infection for subjective diagnosis of VAP. Under strong pressure on hospitals to minimize VAP, these subjective criteria have been applied with increasing stringency, resulting in progressively lower prevalence of VAP. Indeed, previous NHSN reports indicate zero prevalence of VAP in more than 50 % of non-teaching ICUs in the USA [8, 9]. To a certain extent, this decrease reflects artifacts of VAP surveillance methods rather than true improvements in care [10].

As VAP surveillance has limited accuracy, the Centers for Disease Control (CDC) recommended a new surveillance paradigm based on ventilation-associated events (VAE) to assess complications in patients receiving MV. The ventilator-associated event paradigm includes a hierarchy of surveillance targets - ventilation-associated condition (VAC), infection-related ventilated-associated condition (IVAC), and possible and probable VAP. VAC is defined as at least two calendar days of stable or decreasing daily minimum positive end-expiratory pressure (PEEP) or daily minimum fraction of inspired oxygen (FiO_2_) followed by an increase in daily minimum PEEP by at least 3 cm H_2_O sustained for at least two calendar days or an increase in daily minimum FiO_2_ by at least 20 points sustained for at least two calendar days. IVAC is the subset of VAC that may be infection-related based on concurrent inflammatory signs and at least 4 days of new antibiotics. Possible VAP requires either Gram stain evidence of purulence or a pathogenic culture; probable pneumonia requires Gram stain evidence of purulence and quantitative or semi-quantitative growth of a pathogenic organism beyond defined thresholds [11].

The VAE paradigm broadens the focus of surveillance beyond the infectious etiology of respiratory failure to other physiological changes associated with suboptimal ventilator care or progression of underlying diseases, such as pulmonary edema, acute respiratory distress syndrome (ARDS), atelectasis, mucus plugging, pulmonary embolus, and radiation pneumonitis [12].

The NHSN replaced VAP surveillance with VAE surveillance in 2013, because the VAE paradigm makes surveillance more objective to facilitate automation and comparability [10]. Although VAE surveillance shifts the focus away from pneumonia and toward common complications that occur in critically ill patients receiving mechanical ventilation, VAP continues to play a major role in morbidity and length of stay (LOS) and is an important component of VAE. However, whether there are differences between VAP identified by the new VAE surveillance method compared with conventional VAP surveillance remains controversial. Some researchers report good correlation between the two surveillance paradigms [13], while others have claim that VAE surveillance does not accurately reflect VAP [14, 15].

Understanding the difference between VAE and VAP surveillance is valuable, because the change of surveillance paradigm may ultimately affect strategies for VAP prevention and control. Accordingly, we conducted a systematic review and meta-analysis of studies reporting consistency between VAE and VAP. Our objectives were primarily to quantitatively determine the consistency of VAP identification between the two surveillance paradigms, and secondarily to explore the differences in population characteristics between VAE and VAP surveillance.

## Methods

### Selection of studies

We electronically searched literature that reported prevalence of or risk factors for VAE in the PubMed, EMBASE, ScienceDirect, and Cochrane Database on 2 February 2016 for original articles published after 1 January 2010 in peer-reviewed journals. Relevant articles were identified according to the following Boolean expression: (ventilator-associated events [Title/Abstract] OR ventilator-associated conditions [Title/Abstract] OR ventilator-associated complications [Title/Abstract]) AND (prevalence [Mesh] OR risk factors [Mesh]). A reference list of key reviews was also searched for additional studies.

### Selection criteria

Studies that assessed VAE, including VAC, IVAC, possible VAP, and probable VAP, among adult patients who received mechanical ventilation in an ICU were included in our meta-analysis. We included eligible studies that met at least one of the following criteria:

1. Studies providing original data that could be used to calculate the prevalence rate of VAE, odds ratio (OR), or weighted mean difference (WMD) of risk factors for VAE compared to VAP.
2. Studies reporting VAE and VAP in the same population that could be used to calculate relevant indicators of VAE surveillance for the detection of VAP (sensitivity, specificity, positive predictive value (PPV), and negative predictive value (NPV).

Studies of paediatric patients or patients from the emergency department were excluded in our analysis. Conference proceedings, reviews, editorials, commentaries, letters and publications in abstract form only were also excluded. In the case of duplicate studies involving the same subject, we chose the most recent one study.

### Study identification

All titles and abstracts of the citations that were generated by the literature search were screened independently by two reviewers. Relevant publications were reviewed in their entirety, and the reviewers were blinded to the author and research institution of each study. Each reviewer made a judgment on the inclusion or exclusion of the study. In the event of disagreement, a third reviewer served as a consultant to resolve the issue.

### Data extraction

For each included report, the following data were extracted: publication date, region, population, baseline period, hospital, type of ICU, prevalence of VAE with number of cases (*n*) or corresponding denominators (*N*), and risk factors (including age, gender, the acute physiology and chronic health evaluation (APACHE) score, hospital length of stay, ICU length of stay, duration of ventilation, in-hospital mortality, and ICU mortality).

### Quality assessment

The quality of the studies was assessed independently, using the Newcastle–Otawa scale (NOS) for non-randomised observational studies [16], while the Jadad scale was used for randomised controlled trials (RCTs) [17]. The NOS scale allocates a maximum of nine stars to a study, judged on three broad perspectives: the selection of the study groups; the comparability of the groups; and the ascertainment of either the exposure or outcome of interest for case–control or cohort studies, respectively. Studies were defined as poor (0–3), fair (4–6), or good (7–9). The Jadad scale assesses the quality of RCTs relevant to random assignment, double-blinding, and the flow of patients. It allocates a maximum of 5 points to a study. Studies were defined as poor (0–1), fair (2–3), or good (4–5). Two assessors independently evaluated the methodological quality of included studies, and disagreement was resolved through discussion with a third assessor.

### Outcome measures

The primary outcomes were pooled prevalence rate and pooled consistency between VAE and VAP (sensitivity, specificity, PPV, and NPV). The secondary outcomes were pooled ORs and WMDs of relevant factors for VAE compared with VAP (age, sex, APACHE score, LOS, ventilation duration, and mortality). The meta-analysis comparison between VAE and VAP was limited to studies that reported VAE and VAP simultaneously. Studies that reported VAE only were not included in the comparison analysis but were included in the prevalence analysis. In the comparison analysis, continuous data were expressed as WMD and dichotomous data as OR.

### Statistical analyses

A random effects model was used to calculate pooled estimates and their 95 % confidence intervals (CIs) if there was significant heterogeneity among studies. Otherwise, a fixed effects model was chosen. The VAP detection capability was assessed by receiver operating characteristic (ROC) curves. A ROC curve was plotted using the sensitivity and 1− specificity of each study that reported original data. Heterogeneity was assessed by the *Q* test and *I* ^2^ statistic. Egger’s test was used to estimate publication bias in meta-analyses containing more than two individual studies.

Sensitivity analysis was performed by limiting the meta-analysis to studies that used the standard CDC/NHSN definition of VAE for diagnosing VAE and the CDC/NHSN criteria for VAP with quantitative culture results in the diagnosis of VAP, in order to test the impact of the diagnosis method on the pooled results. All tests were two-tailed and statistical significance was defined by a *p* value <0.05. All analyses were conducted using STATA software (version 11.0, Stata corp., College Station, TX, USA). The study was reviewed and approved by the ethical committee of Union Hospital, Tongji Medical College, Huazhong University of Science and Technology.

## Results

**Fig. 1:**
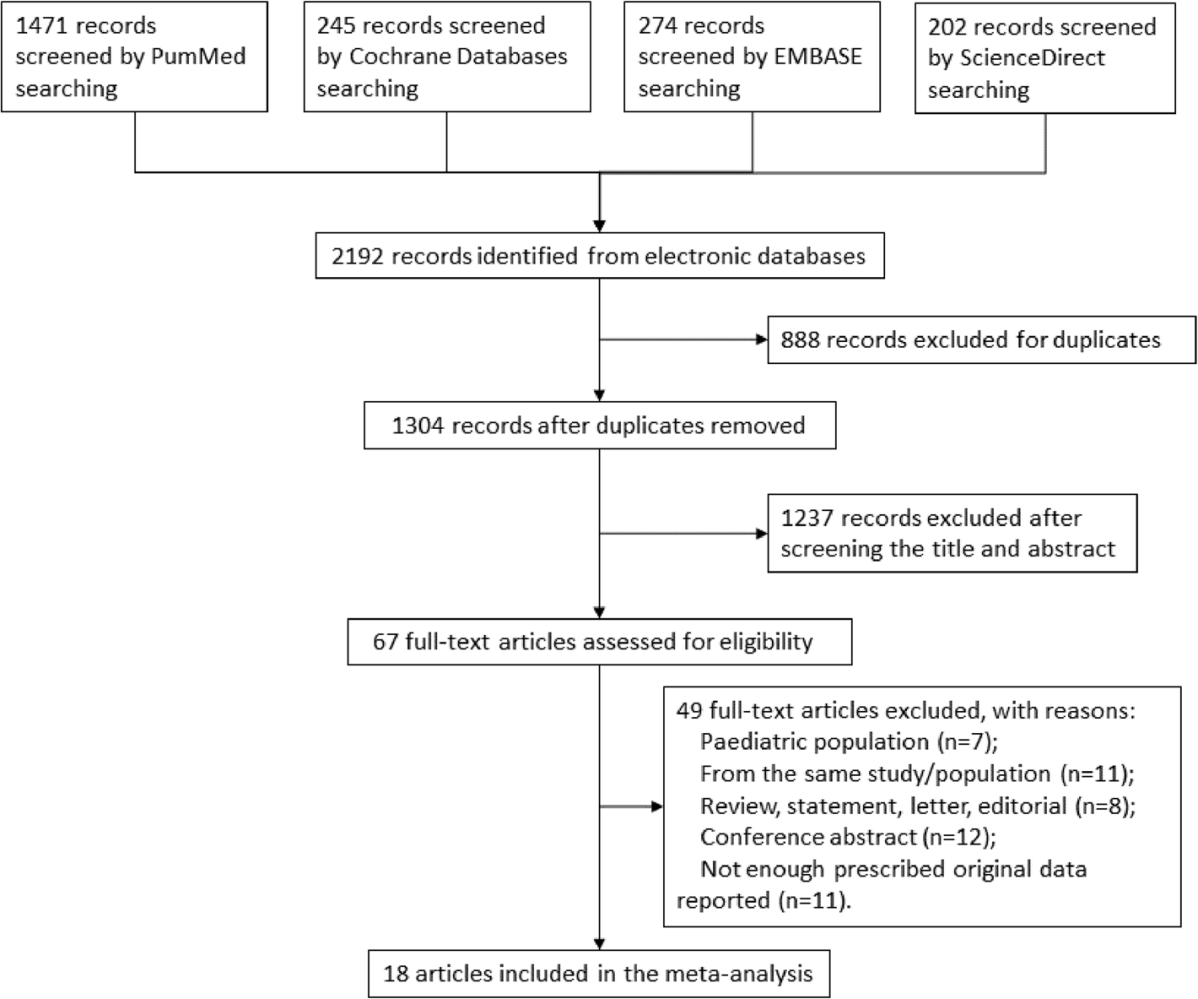
Flow diagram of the selection process

Our search identified 2192 publications. A flow diagram of the selection process is presented in Fig. 1. A total of 888 duplicate publications were removed, and of the remaining 1304 original articles, 1237 were excluded as irrelevant to the study objectives based on their titles and abstracts. Two authors independently reviewed 67 full-text articles and excluded 49 articles that did not meet the selection criteria. Ultimately, 18 studies [18–35] (12 cohort studies, 2 nested case–control studies, 2 time-series analysis studies, 1 screening test, and 1 RCT) were selected for final analysis. One study reported a group of patients from collaborative units undergoing daily spontaneous awakening and spontaneous breathing trials and a group of patients from surveillance-only units [18], and another study reported a group of patients undergoing subglottic secretion suctioning and a group of patients not having subglottic secretion suctioning [19]. For the purpose of our analysis, these groups were treated as four separate studies. Table 1 presents a list of the included studies and their characteristics. In all, the meta-analysis included 61,489 patients who received mechanical ventilation in ICUs in eight countries. Most studies were of acceptable quality, apart from one that was rated as poor (Additional file 1: Table S1).

**Table 1:** Characteristics of studies included in the meta-analysis

Reference	Region	Units (*n*)	ICU type	Baseline period	Population	VAE criteria	VAP criteria	Design	Sample size			Adjusted confounders
									Total (*n*)	VAE	VAP	
[17]^a^	USA	7	MICU, SICU, CICU	2011.11–2013.05	Consecutive mechanical ventilation episodes from collaborative units undergoing daily SAT/SBT	CDC/NHSN definition	NA	Interrupted time series analysis	3425	293	NA	Age, sex, reason for intubation, and SOFA score
[17]^b^	USA	6	MICU, SICU, CICU	2011.11–2013.05	Consecutive mechanical ventilation episodes from surveillance-only units not undergoing daily SAT/SBT	CDC/NHSN definition	NA	Interrupted time series analysis	1739	75	NA	Age, sex, reason for intubation, and SOFA score
[18]^a^	Belgium	1	ICU	2012.01–2013.03	Adult patients ventilated for ≥2 calendar days from groups undergoing subglottic secretion suctioning	CDC/NHSN definition	CDC/NHSN criteria and quantitative culture results of specimens	Randomized controlled trial	170	37	15	NA
[18]^b^	Belgium	1	ICU	2012.01–2013.03	Adult patients ventilated for ≥2 calendar days from groups not undergoing subglottic secretion suctioning	CDC/NHSN definition	CDC/NHSN criteria and quantitative culture results of specimens	Randomized controlled trial	182	41	32	NA
[19]	China	1	ICU	2010.04–2014.02	VAP patients	CDC/NHSN definition	CDC/NHSN criteria	Retrospective cohort	165	55	165	NA
[20]	USA	1	SICU, MICU	2013.01–2013.12	Adult patients ventilated for ≥2 calendar days	CDC/NHSN definition	CDC/NHSN criteria and quantitative culture of specimens	Prospective cohort	1209	67	84	NA
[21]	France	Multiple centres	ICU	1996.11–2012.10	Adult patients ventilated for ≥5 calendar days from French multicentre OUTCOMEREA database	Adapted definition (≥2 day rise in range of PEEP or a decreasing PaO2/FiO2 ratio by >50 mm Hg with the same level of PEEP or by >100 mm Hg whatever the level of PEEP)	CDC/NHSN criteria	Inception cohort	3028	2331	816	NA
[22]	USA	1	MICU	2012.12–2013.04	Adult patients requiring mechanical ventilation	CDC/NHSN definition	NA	Retrospective cohort	257	19	NA	NA
[23]	USA	1	SICU	2012.09–2013.08	All intubated patients admitted to SICU	CDC/NHSN definition	Criteria based on clinical pulmonary infection score (CPIS) and quantitative culture of specimens	Prospective screening test	704	37	121	NA
[24]	USA	1	MICU, SICU	2008.07–2013.03	Adult patients ventilated for ≥4 calendar days continuously	CDC/NHSN definition	CDC/NHSN criteria	Retrospective cohort	3302	675	30^c^	Race, comorbidities, emergent admissions
[25]	USA	1	MICU, SICU, CICU, Neuro-ICU	2009.01–2012.04	Adult patients who were managed with mechanical ventilation	CDC/NHSN definition	CDC/NHSN criteria	Prospective cohort	8408	387	83	APACHE score, type of ICU
[26]	England	1	MICU, SICU,CICU, Neuro-ICU	2011.01–2011.12	Patients on mechanical ventilation in ICU	CDC/NHSN definition	NA	Retrospective nested case–control	2990	172	NA	Age, sex, ICU type, Charlson score, and time to VAC onset
[27]	Netherland	2	ICU	2011.01–2012.07	Adult patients who had received ≥2 consecutive days of mechanical ventilation	CDC/NHSN definition	CDC/NHSN criteria and quantitative culture of specimens	Prospective cohort	2080	152	3	Age, sex, APACHE score, admission type, and hospital
[28]	England	1	ICU	2006.07–2013.12	All episodes of invasive mechanical ventilation lasting ≥3 calendar days	CDC/NHSN definition	NA	Retrospective cohort	9603	1308	NA	Age, race, sex, calendar year, ICU type, comorbidities, initial laboratory values, medications, procedures
[29]	England	1	MICU, SICU, CICU, Neuro-ICU	2006.01–2011.12	Adult patients initiated on mechanical ventilation	CDC/NHSN definition	NA	Retrospective cohort	20356	1056	NA	Age, sex, unit type, Charlson score, use of vasopressors on the day of intubation, platelet count, total bilirubin, albumin, creatinine, and alanine aminotransferase level
[30]	Canada	1	ICU	2011.07–2012.09	VAP patients received at least 72 h broad-spectrum antimicrobials	CDC/NHSN definition	CDC/NHSN criteria	Retrospective cohort	81	45	81	NA
[31]	Canada, USA	11	MICU, SICU, Trauma ICU	2007.06–2009.12	Adult patients who met the inclusion criteria of age ≥16 years and who were mechanically ventilated for ≥48 h	CDC/NHSN definition	CDC/NHSN criteria	Interrupted time series analysis	1320	139	148	NA
[32]	Australia	1	MICU, SICU	2009.05–2011.01	All patients aged >18 years who were on mechanical ventilation for >48 h	Adapted definition (increases in FiO_2_ by ≥15 % or PEEP by ≥2.5 cm H_2_O lasting ≥2 days after stable or decreasing FiO_2_ or PEEP lasting ≥ 2 days.)	NA	Retrospective cohort	1657	153	NA	NA
[33]	USA	3	MICU, SICU	2006–2007	All patients aged >18 years who were on mechanical ventilation for >48 h	Adapted definition (increases in FiO_2_ by ≥15 % or positive end-expiratory pressure (PEEP) by ≥2.5 cm H_2_O lasting ≥2 days after stable or decreasing FiO_2_ or PEEP lasting ≥ 2 days.)	CDC/NHSN criteria	Retrospective nested case–control	597	135	55	Age, sex, hospital, unit type, and Charlson comorbidity index
[34]	USA	1	ICU	2009.07–2013.12	Patients at least 18 years old, admitted to the ICU after trauma, required endotracheal intubation and mechanical ventilator support for at least 48 h, and received a minimum of 1 unit of packed red blood cell transfusion during their mechanical ventilator support	CDC/NHSN definition	CDC/NHSN criteria	Retrospective cohort	216	31	64	NA

^﻿﻿﻿a,b^Study that ​comprised 2 separate groups within one article. ﻿﻿﻿^c^Patients with ventilator-associated pneumonia (*VAP*) were limited to four ICUs among all nine ICUs in the included study. *NHSN* National Healthcare Safety Network, *MICU* medical intensive care unit, *SICU* surgical intensive care unit, *CICU* cardiac intensive care unit, *VAP* ventilator-associated pneumonia, *VAE* ventilator-associated events (including ventilator-associated conditions (*VAC*), infection-related VAC (*IVAC*), possible VAP, and probable VAP), *SAT/SBT* spontaneous awakening trials/spontaneous breathing trials, *PEEP* positive end-expiratory pressure, *FiO*_*2*_ fraction of inspired oxygen, *CDC* Centers for Disease Control, *NHSN* National Healthcare Safety Network, *SOFA* sequential organ failure assessment, *PaO*_*2*_ partial pressure of oxygen, *﻿NA*﻿ ﻿not available﻿﻿

The pooled prevalence rates of each type of VAE and VAP are shown in Table 2. Among mechanically ventilated patients, the pooled prevalence of VAC (13.8 %, 95 % CI 9.0, 18.6 %) was higher, and that of IVAC (6.4 %, 95 % CI 4.8, 8.1 %) lower, than that of VAP (11.9 %, 95 % CI 9.4, 14.4 %). VAE surveillance detected fewer cases of possible and probable VAP among ventilated patients, with pooled prevalence rates of 1.1 % (95 % CI 0.5, 1.7 %) and 0.9 % (95 % CI 0.6, 1.2 %), respectively. Additionally, the pooled prevalence of VAE and VAP increased with the prolongation of ventilation.

**Table 2:** The results of pooled prevalence of VAE and VAP in included studies

	Studies (*n*)	Sample size, (*n*)	Prevalence (%)	95 % Confidence interval (%)	Heterogeneity		Publication bias^a^		Effect model
					*I* ^2^ (%)	*P*	*t* value (Egger test)	*P*	
VAC									
MV >0 day	17	68747	13.8	9.0, 18.6	99.8	<0.01	1.54	0.15	Random
MV ≥2 days	13	25935	20.1	9.6, 30.6	99.8	<0.01	0.53	0.60	Random
MV ≥3 days	5	19408	26.9	5.0, 48.8	99.9	<0.01	0.69	0.54	Random
MV ≥4 days	4	9349	30.7	−1.5, 62.9	99.9	<0.01	0.05	0.97	Random
MV ≥5 days	1	3028	77.0	75.5, 78.5	NA	NA	NA	NA	NA
IVAC									
MV >0 day	14	55430	6.4	4.8, 8.1	99.0	<0.01	2.15	0.05	Random
MV ≥2 days	10	21558	9.6	5.8, 13.3	99.1	<0.01	1.22	0.26	Random
MV ≥3 days	3	15944	14.8	3.9, 25.7	99.7	<0.01	2.60	0.23	Random
MV ≥4 days	2	5885	20.4	4.0, 36.7	99.6	<0.01	NA	NA	Random
MV ≥5 days	1	3028	28.7	27.0, 30.0	NA	NA	NA	NA	NA
Possible VAP									
MV >0 day	7	46820	1.1	0.5, 1.7	97.2	<0.01	0.98	0.37	Random
MV ≥2 days	4	16205	2.5	0.8, 4.1	98.7	<0.01	1.23	0.34	Random
MV ≥3 days	2	12916	4.5	−0.9, 10.0	99.3	<0.01	NA	NA	Random
MV ≥4 days	1	2857	8.5	7.0, 10.0	NA	NA	NA	NA	NA
MV ≥5 days	0	NA	NA	NA	NA	NA	NA	NA	NA
Probable VAP									
MV >0 day	8	47524	0.9	0.6, 1.2	91.7	<0.01	1.14	0.30	Random
MV ≥2 days	5	16642	1.6	0.8, 2.5	94.9	<0.01	1.05	0.37	Random
MV ≥3 days	2	12916	1.7	1.5, 1.9	0.0	0.378	NA	NA	Fixed
MV ≥4 days	1	2557	2.2	2.0, 3.0	NA	NA	NA	NA	NA
MV ≥5 days	0	NA	NA	NA	NA	NA	NA	NA	NA
VAP									
MV >0 day	10	17914	11.9	9.4, 14.4	99.5	<0.01	3.64	<0.01	Random
MV ≥2 days	10	12552	13.7	9.3, 18.1	99.5	<0.01	3.79	<0.01	Random
MV ≥3 days	2	6341	14.7	−9.2, 38.7	99.9	<0.01	NA	NA	Random
MV ≥4 days	2	5885	14.9	−8.6, 38.5	99.9	<0.01	NA	NA	Random
MV ≥5 days	1	3028	26.9	25.0, 29.0	NA	NA	NA	NA	NA

^a^Egger’s test was used to estimate publication bias in meta-analyses containing more than two individual studies. *VAE* ventilator-associated events including ventilator-associated conditions (VAC), infection-related ventilator-associated conditions (IVAC), and possible ventilator-associated pneumonia (VAP), *MV* mechanical ventilation, ﻿*NA*﻿ ﻿not available﻿﻿

**Fig. 2:**
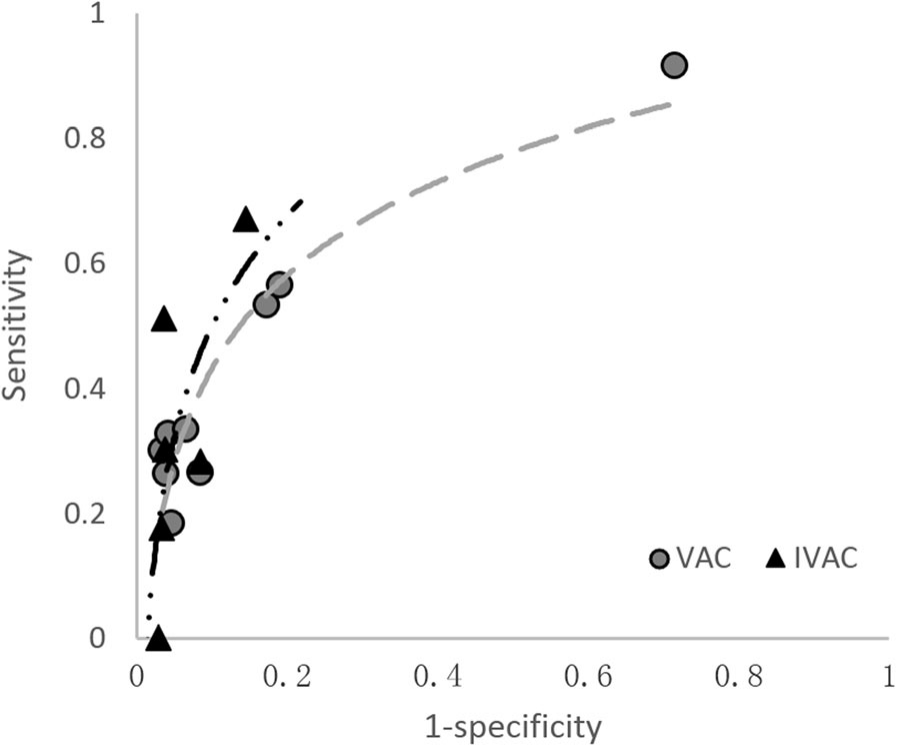
The receiver operating characteristic (ROC) curves for ventilator-associated conditions (*VAC*) and infection-related VAC (*IVAC*) for the detection of ventilator-associated pneumonia (VAP). Scatter points were plotted by the pooled sensitivity and 1-specificity of each included study and trend lines were fitted by the log function. ROCs were not plotted for possible and probable VAP, because studies that provided original sensitivity and specificity data were scarce

In consistency analysis of VAE and VAP, pooled sensitivity was the highest for VAC at 41.8 % (95 % CI 17.7, 65.9 %) and lowest for probable VAP at 1.6 % (95 % CI 0.1, 3.2 %). Pooled PPV was the highest for IVAC at 47.2 % (95 % CI 16.1, 78.3 %) and lowest for probable VAP at 6.5 % (95 % CI 0.3, 12.6 %). Overall, the pooled estimates of sensitivity and PPV of each VAE type for the detection of VAP did not exceed 50 %. By contrast, the pooled specificity and NPV of VAC and IVAC were greater than 80 %, and those of possible VAP and probable VAP were nearly 100 % (Table 3). The ROC curve for IVAC showed a better capability of VAP detection compared with that of VAC (Fig. 2). ROC curves for possible and probable VAP were not plotted, because studies that provided original sensitivity and specificity data were scarce.

**Table 3:** The results of pooled estimates of VAE criteria for the detection of VAP

	Studies (*n*)	Patients (*n*)	Estimates (%)	95 % Confidence interval (%)	Heterogeneity		Publication bias^e^		Effect model
					*I* ^2^ (%)	*P*	*t* value (Egger’s test)	*P*	
Sensitivity									
VAC	11	1633^a^	41.8	17.7, 65.9	99.2	<0.01	−3.53	<0.01	Random
IVAC	6	1323^a^	36.3	14.4, 58.3	98.4	<0.01	−1.74	0.16	Random
Possible VAP	2	248^a^	14.4	10.1, 18.8	70.6	0.07	NA	NA	Fixed
Probable VAP	2	248^a^	1.6	0.1, 3.2	51.9	0.15	NA	NA	Fixed
Specificity									
VAC	9	23112^b^	84.5	76.6, 92.4	99.8	<0.01	−1.50	0.18	Random
IVAC	6	14459^b^	94.0	91.4, 96.7	97.7	<0.01	−0.99	0.38	Random
Possible VAP	1	8325^b^	97.3	96.9, 97.6	NA	NA	NA	NA	NA
Probable VAP	1	8325^b^	99.3	99.1, 99.5	NA	NA	NA	NA	NA
Positive predictive value									
VAC	9	3572^c^	23.2	12.6, 33.9	99.1	<0.01	1.25	0.25	Random
IVAC	5	1563^c^	47.2	16.1, 78.3	99.5	<0.01	0.63	0.57	Random
Possible VAP	1	243^c^	7.4	4.1, 10.7	NA	NA	NA	NA	NA
Probable VAP	1	62^c^	6.5	0.3, 12.6	NA	NA	NA	NA	NA
Negative predictive value									
VAC	9	20927^d^	95.6	94.7, 96.5	98.2	<0.01	−7.30	<0.01	Random
IVAC	6	14211^d^	93.3	91.5, 95.0	99.1	<0.01	−3.89	0.02	Random
Possible VAP	1	8165^d^	99.2	99.0, 99.4	NA	NA	NA	NA	NA
Probable VAP	1	8346^d^	99.1	98.8, 99.3	NA	NA	NA	NA	NA

^a^Number of patients with ventilator-associated pneumonia (*VAP*). ^b^Number of patients without VAP. ^c^Number of patients in the corresponding ventilator-associated events (VAE) type. ^d^Number of patients in the corresponding non-VAE type. ^e^Egger’s test was used to estimate publication bias in meta-analyses containing more than two individual studies, ﻿*NA*﻿ ﻿not available﻿﻿

VAE include ventilator-associated conditions (*VAC*), infection-related ventilated-associated conditions (*IVAC*), possible VAP, and probable VAP

The results of comparisons of population characteristics between VAE and VAP surveillance are shown in Table 4. In-hospital mortality in VAE was higher than that of VAP: the pooled OR of death in hospital was 1.49 (95 % CI 1.11, 2.01) for VAC and 1.76 (95 % CI 1.23, 2.52) for IVAC. Hospital LOS was shorter for VAE compared to VAP: the pooled WMD of hospital LOS was −4.27 days (95 % CI −7.00, −1.55 days) for VAC and −5.86 days (95 % CI −9.46, −2.25 days) for IVAC. Additionally, compared with VAP, the pooled WMD of ventilation duration was −2.79 days (95 % CI −4.79, −0.80 days) for VAC and −2.89 days (95 % CI −5.58, −0.20 days) for IVAC. On the other hand, VAE and VAP did not significantly differ by age, sex, APACHE score, or ICU LOS.

**Table 4:** Risk factors in patients with VAE compared with patients with VAP in all included studies

	Studies (*n*)	Patients with VAE (*n*)	Patients with VAP (*n*)	Estimate (OR/WMD)	95 % Confidence interval (%)	Heterogeneity		Publication bias^c^		Effect model
						*I* ^2^ (%)	*P*	*t* value (Egger’s test)	*P*	
VAC										
Age	1	37	121	0.00^a^	−5.89, 5.89	NA	NA	NA	NA	NA
Sex (male/female)	3	551	255	0.85^b^	0.59, 1.21	20.60	0.28	−2.68	0.24	Fixed
APACHE	1	387	83	5.00^a^	−1.67, 11.67	NA	NA	NA	NA	NA
Ventilated duration (days)	3	569	299	−2.79^a^	−4.79, −0.80	0.00	0.81	−3.39	0.18	Fixed
Death in hospital	5	857	393	1.49^b^	1.11, 2.01	53.10	0.07	−1.37	0.27	Fixed
Length of stay (days)										
In hospital	4	1244	329	−4.27^a^	−7.00, −1.55	47.40	0.13	0.20	0.94	Fixed
In ICU	2	802	81	−0.64^a^	−5.84, 4.55	74.50	0.05	NA	NA	Random
IVAC										
Age	1	31	121	0.00^a^	−6.63, 6.63	NA	NA	NA	NA	NA
Sex (male/female)	3	406	268	1.05^b^	0.70, 1.59	0.00	0.46	0.29	0.82	Fixed
APACHE	1	344	83	4.00^a^	−2.53, 10.53	NA	NA	NA	NA	NA
Ventilated duration (days)	2	384	248	−2.89^a^	−5.58, −0.20	0.00	0.80	NA	NA	Fixed
Death in hospital	4	446	423	1.76^b^	1.23, 2.52	0.00	0.77	0.50	0.67	Fixed
Length of stay (days)										
In hospital	2	384	248	−5.86^a^	−9.46, −2.25	0.00	0.92	NA	NA	Fixed
In ICU	0	0	0	NA	NA	NA	NA	NA	NA	NA

^a^Estimate refers to weighted mean difference (*WMD*). ^b^Estimate refers to odds ratio (*OR*). ^c^Egger’s test was used to estimate publication bias in meta-analyses containing more than two individual studies. *VAE* ventilator-associated conditions (including ventilator-associated conditions (*VAC*), infection-related ventilated-associated conditions (*IVAC*), possible ventilator-associated pneumonia (*VAP*), and probable VAP), *APACHE* acute physiology and chronic health evaluation, *﻿NA*﻿ ﻿not available﻿﻿

In sensitivity analysis, limiting the meta-analysis to studies that employed the standard CDC/NHSN criteria for VAE [18–21, 23–32, 35] and definite VAP identified by quantitative culture of specimens from patients [19, 21, 24, 28, 35], the pooled estimates were robust except for pooled prevalence (Additional file 1: Tables S2–S4). The pooled prevalence rates of each VAE type decreased but VAP increased after limiting the analyses to these studies. The new pooled prevalence of VAC (8.0 %, 95 % CI 6.5, 9.6 %) and IVAC (4.0 %, 95 % CI 3.1, 4.9 %) were lower than that of VAP (13.0 %, 95 % CI 6.3, 19.7 %).

Among all meta-analyses containing more than two individual studies, publication bias was detected only for pooled sensitivity (*p* < 0.01) and negative predictive value of VAC (*p* < 0.01), and pooled prevalence of VAP (*p* = 0.01).

## Discussion

In our systematic review, the pooled VAE prevalence among patients who received mechanical ventilation in the ICU, 13.8 %, was higher than the observed 11.9 % pooled prevalence of VAP. This result is reasonable, because the VAE paradigm aims to identify a broader range of ventilator-associated complications, including both infectious complications (such as pneumonia, tracheitis, and tracheobronchitis) and non-infectious complications (such as atelectasis, pulmonary embolism, pulmonary oedema, and ventilator-induced lung injury) [36]. In a previous study VAP was estimated to be the most common complication, accounting for about 25–40 % of VAE, followed by fluid overload at 20–40 %, ARDS at 10–20 %, and atelectasis at 10–15 % [37]. Theoretically, by excluding non-infectious complications among VAE, IVAC should be more representative of VAP, and its prevalence should be closer to but still higher than VAP. However, in our meta-analysis, the pooled prevalence of IVAC was lower than that of VAP, and even in the sensitivity analysis, both VAC and IVAC were lower than VAP after limiting the evaluation to studies that used stricter diagnostic criteria. This result indicates that VAE surveillance might miss a certain number of cases of VAP.

Actually, among the 11 studies included in our meta-analysis that reported both VAC and VAP [19–22, 24–26, 28, 31, 32, 34], the pooled sensitivity of VAC for the detection of VAP was not satisfactory. Only 41.8 % of cases of VAP could be identified by using the VAC criteria; in other words, VAC surveillance missed about 60 % of ventilated patients who developed pneumonia. Similarly, the pooled PPV of VAC from nine studies [19, 21, 22, 24–26, 28, 32, 34] also indicated a poor capability for VAP detection. Only 23.2 % of patients who met the VAC criteria would be diagnosed as having VAP. Even excluding the non-infectious complications among VAE, the pooled sensitivity and PPV of IVAC, possible VAP and probable VAP for the detection of VAP were still low.

Most patients with VAP did not meet the VAE criteria, mainly because they did not meet the requirements for stable baseline mechanical ventilator settings or threshold levels of worsening gas exchange. In a study by Lilly et al., 70.8 % of patients with VAP did not have 2 days of stable oxygenation in the time frame required by the VAE criteria [26]. Moreover, this percentage was 82.6 % in a study by Annop at al., and only 17.4 % for insufficient levels of worsening gas exchange [35]. In contrast, in the study by Klouwenberg at al. [28], among VAP episodes that did not fulfil the criteria for VAE, those with no baseline period of stability accounted for only 24.0 % of the cohort, and those with insufficient increase in ventilator settings accounted for 76.0 % of the cohort. Similarly, in a study by Stoeppel et al., these rates were 39.4 % for insufficient period of stability followed by worsening oxygenation and 47.5 % for duration of respiratory deterioration less than 2 days [24].

Another explanation for the poor validity of VAE criteria for identifying VAP is that VAE criteria do not rely on chest radiography, which is the most sensitive indicator of pathologically diagnosed VAP [38, 39]. On the other hand, although the established diagnostic criteria for VAP are widely clinically accepted and applied, recent VAP criteria are seriously flawed in the subjectivity of clinical diagnosis, which might be another factor affecting the consistency of detection results between the two surveillance methods [40].

In our meta-analysis, although there were high rates of pooled specificity and high NPV for the VAE paradigm, the value of VAE for VAP detection was limited, given that negative screens are caused not only by cases in which the entity is absent, but also by those in which the entity is missed or not clear. In fact the VAP diagnostic criteria, which have been regarded as the gold standard for screening tests, are not objective or specific criteria, so high specificity or NPV for VAE does not indicate that an acceptable proportion of cases of VAP were detected by the screening test.

Some characteristics of the populations identified by VAE and VAP surveillance also significantly differed. Both VAE and VAP could prolong the length of ventilation and hospital stay, but the risk intensity was different in these two paradigms. By definition, VAE and VAP should differ in ventilation duration, because VAP requires the patient to receive mechanical ventilation for more than 2 days, while at least 4 days are required for VAE. However, interestingly, the pooled WMD of ventilation duration for VAE was about −3 days compared with VAP. This result implies that patients with VAE who did not meet VAP criteria such as fluid overload, ARDS, and atelectasis, tended to receive a shorter duration of mechanical ventilation. Similarly, the mean hospital LOS for VAC and IVAC was about 4–6 days shorter than that of VAP. These differences may have been due to discrepancies in the severity of comorbidities and differences in the timing of extubation.

Additionally, the in-hospital mortality of VAC and IVAC was approximately twofold higher than that of VAP. In fact, a sustained decrease in oxygenation is an independent risk factor for mortality in ventilated patients [41], and thus, higher mortality associated with VAE may due to the VAE criteria aiding in the detection of more severe patients with poorer oxygenation [20]. Only patients with complications severe enough to merit the threshold levels of worsening gas exchange met the VAE criteria, whereas patients with slight worsening of gas exchange could still be diagnosed with VAP [34]. Indeed, about 60 % of patients diagnosed with VAP did not meet the VAE criteria in our meta-analysis.

Overall, we found that patients’ duration of ventilation and hospital stay were shorter in the VAE paradigm than in the VAP paradigm, while in-hospital mortality was higher in the VAE paradigm than in the VAP paradigm. In other words, the characteristics of patients identified by VAP surveillance were not accurately reflected by VAE surveillance. Confounding complications in VAE cases could have an influence on the significance of risk factors. For example, ARDS was the most common complication in VAE (46.8 %) in the study of Lilly et al. [26] while it only accounted for 16.4 % of patients with VAE in the study of Boyer et al. [21]. Consequently, the OR of in-hospital death for VAE compared with VAP in the former study was 1.50 (95 % CI 0.88 ~ 2.56) with no statistical difference, whereas it was 2.29 (95 % CI 1.19 ~ 4.43) reflecting a statistical difference in the latter study. The difference in distributions of complications may affect the population characteristics of VAE surveillance, which aims to identify a broader spectrum of complications of mechanical ventilation.

VAE surveillance has several advantages. First, VAE diagnosis is less time-consuming than traditional VAP diagnosis. The VAE paradigm was designed to rely on objective measures that can be easily assessed by professionals in the detection of infection. A study conducted in two hospitals indicated that VAE reviewers required 12 h to manually diagnose 400 ventilated patients, while the traditional VAP reviewer required 260 hours [34]. Furthermore, objective measures can be easily coded into computerized programmes. Consequently, software-based automatic data collection processes can further reduce the time needed for VAE identification. In a previous study, automatic VAE surveillance required only 1 minute to assess 110 patients, compared to 60.7 minutes using manual surveillance [42].

Second, the VAE paradigm maximizes the objectivity of surveillance to improve comparability [10]. Quantitative measures of VAE are commonly available in every ICU; thus, the objective criteria enable different institutions to compare their rates with greater confidence, such that differences in rates reflect differences in patients and processes of care rather than subjective and unquantifiable surveillance biases.

Third, including a broader spectrum of complications is beneficial to identifying a population of patients with serious complications who have not been acknowledged previously [2]. In fact, the VAE paradigm is able to identify not only patients with complications of mechanical ventilation but also those with severe respiratory compromise or progressive underlying disease, despite optimal care. Therefore, the broader spectrum is beneficial to monitoring critically ill patients in the ICU, making it possible to prevent severe complications at an early stage.

Strengths of our study include the quantitative methodology of the systematic review, a large sample size for estimating the prevalence of each type of VAE, and the assessment of consistency between VAE and VAP surveillance. However, our meta-analysis also has limitations. First, heterogeneity is a common problem for meta-analyses of observational studies, particularly those that involve proportions [43–45]. We attempted to explain heterogeneity by performing subgroup analyses, but after an exploration of the factors that were likely to contribute to the variation, such as study design and population characteristics, the heterogeneity remained unexplained. Although objective measures were used as the criteria for VAE monitoring, they were implemented independently in different hospitals and ICUs. Therefore, it is difficult to ensure that all surveillance activities are homogeneous in clinical practice. These constraints and variations in setting, such as patients’ baseline characteristics, data collection methods, and surveillance systems, may account at least in part for the significant heterogeneity observed [28].

Second, not every study reported prevalence or the original number of cases of VAE within each ventilation duration group, which is why the number of included studies within each ventilation duration sub-group was not equal, particularly for the small number of studies in groups with MV ≥3 days, MV ≥4 days, and MV ≥5 days. The insufficient number of studies included in these groups led to an unstable pooled prevalence with a broader 95 % CI in the meta-analysis; even 95 % CIs in which the lower limit was negative were observed in several groups in our study. Therefore, the results in these groups should be interpreted with caution, and more high-quality studies with standardized ventilation duration groups are needed in the future.

Third, we did not conduct sub-group analysis in different types of ICU because of insufficient sub-group data within each type. The type of ICU type be a significant confounding factor affecting the prevalence and population characteristics of patients with VAE and VAP [26, 30]. Although the studies included in the meta-analysis reported their own ICU type, most provided the overall data from mixed ICU types rather than the sub-group data for each type. Consequently, it was difficult to separate total data into sub-group data according to ICU type in most included studies.

Finally, not all studies in our meta-analysis used the same diagnostic criteria. We included three studies that did not strictly meet CDC/NHSN criteria for VAE. A multicentre study in France slightly adapted the VAE definition, taking into account the change in PaO_2_/FiO_2_ with regard to the level of PEEP as a more reliable criterion for the assessment of worsening oxygenation [22]. Two studies used an early definition of VAE prior to the implementation of VAE surveillance by the NHSN in January 2013 [33, 34]. These early studies diagnosed VAE using looser criteria relative to the CDC/NHSN definition. In addition, our meta-analysis contains five studies that applied stricter VAP diagnostic criteria with quantitative culture of specimens [19, 21, 24, 28, 35]. After limiting the meta-analysis to these studies using stricter diagnostic criteria, the pooled estimates of each VAE type and of VAP changed, but these changes have limited effects on the relationship between VAE and VAP: the VAE paradigm still missed a certain number of cases of VAP, in-hospital mortality was still higher in patients with VAE than with VAP, and ventilation duration was still shorter in patients with VAE than with VAP.

## Conclusions

Overall, the findings of the present study indicate that VAE surveillance may not be suitable for identifying patients with VAP. As each surveillance paradigm has its own advantages, we suggest that traditional VAP surveillance should not be replaced entirely by VAE surveillance, but rather for both VAE and VAP surveillance to be carried out in tandem according to the specific conditions of each hospital and ICU.

## Additional file

Additional file 1: Table S1. Quality assessment of included studies. **Table S2.** Results for analysis of pooled prevalence of ventilator-associated events and ventilator-associated pneumonia in the sensitivity analysis. **Table S3.** Results of pooled estimates of ventilator-associated events criteria for the detection of ventilator-associated pneumonia in the sensitivity analysis. **Table S4.** Risk factors for patients with ventilator-associated events compared with patients with ventilator-associated pneumonia in the sensitivity analysis. (DOCX 32 kb)

## Abbreviations

APACHE: acute physiology and chronic health evaluation
ARDS: acute respiratory distress syndrome
CDC: the Centers for Disease Control
CIs: confidence intervals
CPIS: the clinical pulmonary infection score
FiO_2_: fraction of inspired oxygen
ICU: intensive care unit
IVAC: infection-related ventilator-associated conditions
LOS: length of stay
MV: mechanical ventilation
NHSN: the National Healthcare Safety Network
NPV: negative predictive value
OR: odds ratio
PEEP: positive end-expiratory pressure
PPV: positive predictive value
RCT: randomized control trial
ROC: receiver operating characteristic
VAC: ventilator-associated conditions
VAE: ventilator-associated event
VAP: ventilator-associated pneumonia
WBC: white blood cells
WMD: weighted mean difference
