# Supplementary material for: Does ventilator-associated event surveillance detect ventilator-associated pneumonia in intensive care units? A systematic review and meta-analysis
Source: Crit Care. 2016 Oct 24;20:338. doi: 10.1186/s13054-016-1506-z (PMC5075751; doi:10.1186/s13054-016-1506-z)
Supplement: Additional file 1: Table S1. — Quality assessment of included studies. Table S2. Results for analysis of pooled prevalence of ventilator-associated events and ventilator-associated pneumonia in the sensitivity analysis. Table S3. Results of pooled estimates of ventilator-associated events criteria for the detection of ventilator-associated pneumonia in the sensitivity analysis. Table S4. Risk factors for patients with ventilator-associated events compared with patients with ventilator-associated pneumonia in the sensitivity analysis. (DOCX 32 kb) [file 13054_2016_1506_MOESM1_ESM.docx]

| Table S1. The quality assessment for included studies. | | | | | | | | | | |
| --- | --- | --- | --- | --- | --- | --- | --- | --- | --- | --- |
| For cohort and case-control (NOS scale) | Selection | | | |  | Comparability |  | Outcome | | |
|  | 1 | 2 | 3 | 4 |  | 1 |  | 1 | 2 | 3 |
| 17 | ★ | ★ | ★ | / |  | ★ ★ |  | ★ | ★ | / |
| 19 | / | ★ | ★ | / |  | / |  | ★ | ★ | / |
| 20 | ★ | ★ | ★ | / |  | / |  | ★ | ★ | / |
| 21 | / | ★ | / | / |  | / |  | ★ | ★ | / |
| 22 | ★ | / | ★ | / |  | / |  | ★ | ★ | / |
| 23 | ★ | ★ | / | / |  | / |  | ★ | ★ | / |
| 24 | ★ | ★ | ★ | / |  | ★ |  | ★ | ★ | / |
| 25 | ★ | ★ | ★ | / |  | ★ |  | ★ | ★ | / |
| 26 | ★ | ★ | / | / |  | ★ ★ |  | ★ | ★ | / |
| 27 | ★ | ★ | ★ | ★ |  | ★ ★ |  | ★ | ★ | / |
| 28 | ★ | ★ | ★ | / |  | ★ ★ |  | ★ | ★ | / |
| 29 | ★ | ★ | ★ | / |  | ★ ★ |  | ★ | ★ | / |
| 30 | / | ★ | ★ | ★ |  | / |  | ★ | ★ | / |
| 31 | ★ | ★ | ★ | / |  | / |  | ★ | ★ | / |
| 32 | ★ | ★ | / | ★ |  | / |  | ★ | ★ | / |
| 33 | ★ | ★ | / | / |  | ★ ★ |  | ★ | ★ | / |
| 34 | ★ | ★ | ★ | ★ |  |  |  | ★ | ★ |  |
| For RCT  (Jadad scale) | Randomi-zation  (0~2) | | Double blinding  (0~2) | |  | Concealment of allocation  (0~2) |  | Withdrawals and dropouts  (0~1) | | |
| 18 | ★ ★ | | / | |  | ★ ★ |  | ★ | | |

| Table S2. The results of pooled prevalence of ventilator-associated events and ventilator-associated pneumonia in the sensitivity analysis | | | | | | | | | | | |
| --- | --- | --- | --- | --- | --- | --- | --- | --- | --- | --- | --- |
| Group | No. of studies | Sample size | Prevalence (%) | 95% confidence interval (%) |  | Heterogeneity | |  | Publication bias^@^ | | Effect model |
|  |  |  |  |  |  | I^2^ (%) | *P* |  | t value of Egger's test | *P* |  |
| VAC |  |  |  |  |  |  |  |  |  |  |  |
| MV>0day | 14 | 63465 | 8.0 | 6.5 ~ 9.6 |  | 98.0 | <0.01 |  | 1.56 | 0.14 | random |
| MV≥2days | 10 | 29474 | 12.9 | 9.8 ~15.9 |  | 97.5 | <0.01 |  | 0.11 | 0.92 | random |
| MV≥3days | 4 | 16380 | 14.6 | 10.8 ~ 18.3 |  | 97.1 | <0.01 |  | 0.30 | 0.79 | random |
| MV≥4days | 3 | 6321 | 15.5 | 9.9 ~ 21.1 |  | 96.5 | <0.01 |  | 0.17 | 0.89 | random |
| MV≥5days | 0 | / | / | / |  | / | / |  | / | / | / |
| IVAC |  |  |  |  |  |  |  |  |  |  |  |
| MV>0day | 13 | 52402 | 4.0 | 3.1 ~ 4.9 |  | 95.9 | <0.01 |  | 2.24 | 0.05 | random |
| MV≥2days | 9 | 18530 | 6.9 | 5.0 ~ 8.7 |  | 95.5 | <0.01 |  | 1.02 | 0.34 | random |
| MV≥3days | 2 | 12916 | 7.9 | 3.0 ~ 12.7 |  | 98.6 | <0.01 |  | / | / | random |
| MV≥4days | 1 | 2857 | 12.4 | 10.8 ~ 13.2 |  | / | / |  | / | / | / |
| MV≥5days | 0 | / | / | / |  | / | / |  | / | / | / |
| Possible VAP |  |  |  |  |  |  |  |  |  |  |  |
| MV>0day | 7 | 46820 | 1.1 | 0.5 ~ 1.7 |  | 97.2 | <0.01 |  | 0.98 | 0.37 | random |
| MV≥2days | 4 | 16205 | 2.5 | 0.8 ~ 4.1 |  | 98.7 | <0.01 |  | 1.23 | 0.34 | random |
| MV≥3days | 2 | 12916 | 4.5 | -0.9 ~ 10.0 |  | 99.3 | <0.01 |  | / | / | random |
| MV≥4days | 1 | 2857 | 8.5 | 7.0 ~ 10.0 |  | / | / |  | / | / | / |
| MV≥5days | 0 | / | / | / |  | / | / |  | / | / | / |
| Probable VAP |  |  |  |  |  |  |  |  |  |  |  |
| MV>0day | 8 | 47524 | 0.9 | 0.6 ~ 1.2 |  | 91.7 | <0.01 |  | 1.14 | 0.30 | random |
| MV≥2days | 5 | 16642 | 1.6 | 0.8 ~ 2.5 |  | 94.9 | <0.01 |  | 1.05 | 0.37 | random |
| MV≥3days | 2 | 12916 | 1.7 | 1.5 ~ 1.9 |  | 0.0 | 0.378 |  | / | / | fixed |
| MV≥4days | 1 | 2557 | 2.2 | 2.0 ~ 3.0 |  | / | / |  | / | / | / |
| MV≥5days | 0 | / | / | / |  | / | / |  | / | / | / |
| VAP |  |  |  |  |  |  |  |  |  |  |  |
| MV>0day | 6 | 4561 | 13.0 | 6.3 ~ 19.7 |  | 98.6 | <0.01 |  | 5.57 | <0.01 | random |
| MV≥2days | 6 | 4294 | 14.8 | 7.5 ~ 22.0 |  | 98.7 | <0.01 |  | 5.34 | <0.01 | random |
| MV≥3days | 0 | / | / | / |  | / | / |  | / | / | / |
| MV≥4days | 0 | / | / | / |  | / | / |  | / | / | / |
| MV≥5days | 0 | / | / | / |  | / | / |  | / | / | / |
| VAE, Ventilator-associated events including VAC, IVAC, Possible VAP, and Probable VAP. | | | | | | | | | | | |
| VAC, Ventilator-associated conditions  IVAC, Infection-related ventilated-associated condition | | | | | | | | | | | |
| VAP, Ventilator-associated pneumonia  ^@^Egger’s test was used to estimate publication bias in meta-analyses containing more than two individual studies. | | | | | | | | | | | |

| Table S3. The results of pooled estimates of ventilator-associated events criteria for the detection of ventilator-associated pneumonia in the sensitivity analysis | | | | | | | | | | | |
| --- | --- | --- | --- | --- | --- | --- | --- | --- | --- | --- | --- |
| Group | No. of studies | No. of patients | Estimates (%) | 95% confidence interval (%) |  | Heterogeneity | |  | Publication bias^@^ | | Effect model |
|  |  |  |  |  |  | I^2^ (%) | *P* |  | t value of Egger | *P* |  |
| Sensitivity (%) | | | | | | | | | | | |
| VAC | 4 | 255^a^ | 31.5 | 15.4 – 47.6 |  | 84.2 | <0.01 |  | 0.93 | 0.45 | random |
| IVAC | 2 | 111^a^ | 39.1 | 16.7 – 61.6 |  | 83.9 | 0.01 |  | / | / | random |
| Possible VAP | 0 | / | / | / |  | / | / |  | / | / | / |
| Probable VAP | 0 | / | / | / |  | / | / |  | / | / | / |
| Specificity (%) | | | | | | | | | | | |
| VAC | 4 | 4039^b^ | 92.6 | 89.5 - 95.6 |  | 92.7 | <0.01 |  | 1.02 | 0.42 | random |
| IVAC | 3 | 2750^b^ | 96.0 | 93.8 – 98.1 |  | 69.7 | 0.04 |  | 2.37 | 0.25 | random |
| Possible VAP | 0 | / | / | / |  | / | / |  | / | / | / |
| Probable VAP | 0 | / | / | / |  | / | / |  | / | / | / |
| Positive predictive value (%) | | | | | | | | | | | |
| VAC | 4 | 334^c^ | 30.6 | 3.9 - 57.3 |  | 97.4 | <0.01 |  | 14.4 | <0.01 | random |
| IVAC | 2 | 220^c^ | 59.9 | 53.5 - 66.4 |  | 32.3 | 0.22 |  | / | / | fixed |
| Possible VAP | 0 | / | / | / |  | / | / |  | / | / | / |
| Probable VAP | 0 | / | / | / |  | / | / |  | / | / | / |
| Negative predictive value (%) | | | | | | | | | | | |
| VAC | 4 | 3960^d^ | 90.8 | 84.6 - 97.0 |  | 98.6 | <0.01 |  | 4.27 | 0.05 | random |
| IVAC | 3 | 2733^d^ | 89.9 | 79.9 - 99.7 |  | 97.6 | <0.01 |  | 4.70 | 0.13 | random |
| Possible VAP | 0 | / | / | / |  | / | / |  | / | / | / |
| Probable VAP | 0 | / | / | / |  | / | / |  | / | / | / |
| ^a^ the number of VAP patients, ^b^ the number of Non-VAP patients, ^c^ the number of patients in corresponding VAE type,  ^d^ the number of patients in corresponding Non-VAE type. ^@^Egger’s test was used to estimate publication bias in meta-analyses containing more than two individual studies. | | | | | | | | | | | |
| VAE, Ventilator-associated events including VAC, IVAC, Possible VAP, and Probable VAP. | | | | | | | | | | | |
| VAC, Ventilator-associated conditions  IVAC, Infection-related ventilated-associated condition | | | | | | | | | | | |
| VAP, Ventilator-associated pneumonia | | | | | | | | | | | |

| Table S4. Risk factors of ventilator-associated events patients compared with ventilator-associated pneumonia patients in the sensitivity analysis | | | | | | | | | | | | |
| --- | --- | --- | --- | --- | --- | --- | --- | --- | --- | --- | --- | --- |
| Group | No. of studies | No. of VAE patients | No. of VAP patients | Estimate (OR/WMD) | 95% confidence interval (%) |  | Heterogeneity | |  | Publication bias^@^ | | Effect model |
|  |  |  |  |  |  |  | I^2^ (%) | P |  | t value of Egger | P |  |
| VAC |  |  |  |  |  |  |  |  |  |  |  |  |
| Age | 1 | 37 | 121 | 0.00^a^ | -5.89 ~ 5.89 |  | / | / |  | / | / | / |
| Sex (male/female) | 1 | 37 | 121 | 0.51^b^ | 0.23 ~ 1.09 |  | / | / |  | / | / | / |
| APACHE | 0 | / | / | / | / |  | / | / |  | / | / | / |
| Ventilated duration (Day) | 2 | 442 | 248 | -3.22^a^ | -5.82 ~ -0.61 |  | 0.00 | 0.67 |  | / | / | fixed |
| Death in hospital | 2 | 89 | 235 | 1.60^b^ | 0.97 ~ 2.66 |  | 68.2 | 0.07 |  | / | / | fixed |
| Length of stay (Day) |  |  |  |  |  |  |  |  |  |  |  |  |
| In hospital | 0 | / | / | / | / |  | / | / |  | / | / | / |
| In ICU | 0 | / | / | / | / |  | / | / |  | / | / | / |
| IVAC |  |  |  |  |  |  |  |  |  |  |  |  |
| Age | 1 | 31 | 121 | 0.00^a^ | -6.63 ~ 6.63 |  | / | / |  | / | / | / |
| Sex (male/female) | 2 | 62 | 185 | 0.99^b^ | 0.49 ~ 2.02 |  | 31.4 | 0.23 |  | / | / | fixed |
| APACHE | 0 | / | / | / | / |  | / | / |  | / | / | / |
| Ventilated duration (Day) | 2 | 384 | 248 | -2.89^a^ | -5.58 ~ -0.20 |  | 0.00 | 0.80 |  | / | / | fixed |
| Death in hospital | 3 | 102 | 350 | 1.97^b^ | 1.22 ~ 3.18 |  | 0.00 | 0.68 |  | 1.15 | 0.46 | fixed |
| Length of stay (Day) |  |  |  |  |  |  |  |  |  |  |  |  |
| In hospital | 0 | / | / | / | / |  | / | / |  | / | / | / |
| In ICU | 0 | / | / | / | / |  | / | / |  | / | / | / |
| ^a^ the estimate refers to WMD, Weighted Mean Difference , ^b^ the estimate refers to OR, odds ratio. ^@^Egger’s test was used to estimate publication bias in meta-analyses containing more than two individual studies. VAE, Ventilator-associated events including VAC, IVAC, Possible VAP, and Probable VAP. VAC Ventilator-associated conditions, IVAC Infection-related ventilated-associated condition, VAP Ventilator-associated pneumonia, APACHE The acute physiology and chronic health evaluation. | | | | | | | | | | | | |
